# Graphitic Carbon Nitride and IGZO Bio-FET for Rapid Diagnosis of Myocardial Infarction

**DOI:** 10.3390/bios12100836

**Published:** 2022-10-07

**Authors:** Walaa Khushaim, Mani Teja Vijjapu, Saravanan Yuvaraja, Veerappan Mani, Khaled Nabil Salama

**Affiliations:** 1 Sensors Lab, Advanced Membranes and Porous Materials Center, Computer, Electrical and Mathematical Science and Engineering Division, King Abdullah University of Science and Technology (KAUST), Thuwal 23955-6900, Saudi Arabia; 2 Sensor Systems Division, Silicon Austria Labs (SAL), High Tech Campus, 9524 Villach, Austria

**Keywords:** field effect transistor, acute myocardial infarction, graphitic carbon nitride, IGZO, cardiovascular disease

## Abstract

Acute myocardial infarction (AMI), commonly known as a heart attack, is a life-threatening condition that causes millions of deaths every year. In this study, a transistor-based biosensor is developed for rapid and sensitive detection of cardiac troponin-I (cTnI), a diagnostic biomarker of AMI. A biosensing technique based on a field effect transistor (FET), which uses indium gallium zinc oxide (IGZO) as an excellent semiconducting channel, is integrated with nanosheet materials to detect cTnI. Porous carbon nitride (PCN) decorated with gold nanoparticles (Au NPs) is used as a bridge between the solid-state device and the biorecognition element. We demonstrate that this biosensor is highly sensitive and has an experimental limit of detection of 0.0066 ng/mL and a dynamic range of 0.01 ng/mL–1000 ng/mL. This is the first report of a semiconducting metal oxide FET cardiac biomarker sensor combined with PCN for the detection of cTnI. The reported compact microsystem paves the way for rapid and inexpensive detection of cardiac biomarkers.

## 1. Introduction

Cardiovascular diseases (CVDs) are the leading cause of mortality worldwide [1,2]. CVD mortality is estimated to exceed 23.3 million by 2030 and results in massive social loss [3,4]. Acute myocardial infarction (AMI), also known as a heart attack, represents more than 85% of cases of CVD [5,6]. Treatments for AMI have progressed significantly, yet the frequency of heart failure remains high, and the survival rate remains poor [7]. Rapid diagnosis of AMI could significantly increase the heart attack survival rate [8,9]. However, it still takes a minimum of one hour to diagnose a patient with AMI using an ELISA to biomedically identify cardiac biomarkers in the blood [10]. One of the most widely used biomarkers for AMI is cardiac troponin-I (cTnI), which is released into the bloodstream from damaged heart muscle tissues within 1–4 h of the onset of AMI [11]. The cut-off level of serum cTnI is 0.03 ng/mL to diagnose AMI [2]. Rapid detection of cTnI with high sensitivity and accuracy using biosensors is vital to improving the survival rates and outcomes of patients who suffer a heart attack.

Biosensors offer integrated molecular recognition, sensing materials, and fast signal readout and have proven to be one of the most important aspects of analytical instruments [12,13,14]. However, traditional biosensors do not have a sufficiently low detection limit to identify biomarkers such as cTnI. Field effect transistors (FETs) are a promising platform for the detection of a variety of biomarkers [15,16]. FET-based biosensors (Bio-FETs) offer advantageous properties such as compactness, ease of integration with point-of-care diagnostic devices, low-cost production, and ease of use [17]. As a result, many studies have explored the potential of Bio-FETs for the detection of pathological biomarkers [8,15,18]. By utilizing specific biorecognition elements on the conducting channel, Bio-FETs can achieve high sensitivity and selectivity for the corresponding biomarker [19]. The FET channel parameters, such as drain-source current (I_DS_), are influenced by the reaction between the biomarker and the biorecognition element [20]. Nevertheless, the commercial development of FET-based biosensors faces several challenges, especially the integration of the biorecognition elements.

The exceptional properties of indium gallium zinc oxide (IGZO), such as high electrical mobility and superior homogeneity, play a significant role in building thin-film transistor devices [21,22]. IGZO FETs have also attracted significant attention in bioengineering and have been used in biological sensors [23]. To create a glucose sensor, an IGZO metal-semiconductor FET was fabricated with Ru-Si-O on top of the IGZO, followed by the immobilization of the glucose oxidase enzyme [24]. Although there have been multiple reports of IGZO being employed in Bio-FETs, additional materials need to be added to the IGZO surface for passivation in order to ensure selectivity. This necessitates complex fabrication processes, which limit the spectrum of applications and increase fabrication costs.

The use of semiconducting polymers for biosensing applications meets the demand for simple immobilization of biorecognition elements, as well as tunable electrical characteristics. Compared to conventional methods, nanomaterials have the ability to alter the surface chemistry and improve the detection limits and sensitivity of the biosensors [11,25,26]. Two-dimensional (2D) semiconducting porous carbon nitride (PCN) exhibits excellent electronic properties such as a high surface area, structural and surface defects, and the presence of active sites for bio-catalysis [27,28]. Such characteristics make PCN one of the best choices to functionalize Bio-FETs. PCN was shown to be effective as a conducting channel in Bio-FETs for the detection of human immunoglobulin G20 [29].

**Figure 1:**
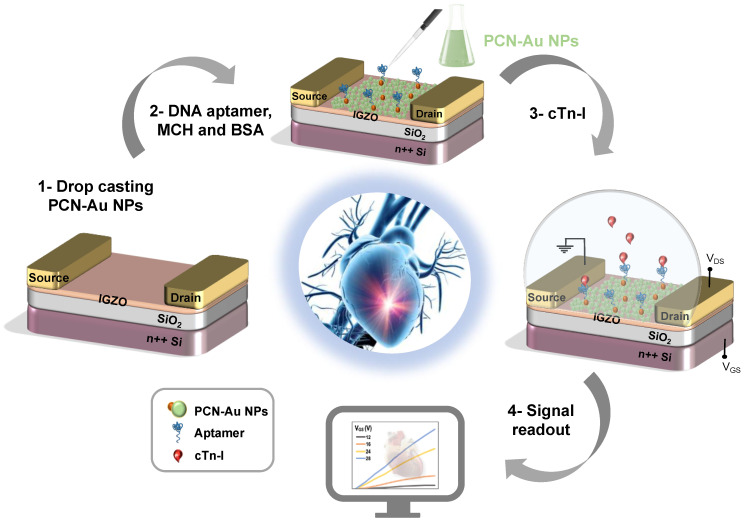
Schematic of the fabricated PCN-Au NPs IGZO TFT aptasensor for cTnI detection (cross-section) with IGZO as the channel, PCN-Au NPs the active sensing layer, and Ti/Au as source and drain electrodes of the transistor.

In this article, we report a sensitivity amplification technique based on the coupling effect of n-type IGZO with PCN material as a receptor-cum-channel layer. While PCN exhibits poor sensitivity toward biomarkers [30], gold nanoparticle (Au NPs)-decorated PCN has a high faradaic nitrogen reduction efficiency, which leads to suitable biocompatibility. Therefore, to overcome the poor sensitivity of the biosensor, we propose a new strategy based on the combination of Au NP-decorated PCN with IGZO FET. PCN-Au NPs provide a bridge between the solid-state device and the biorecognition element, which enhances the immobilization of aptamers and increases the sensitivity and accuracy of the target analyte (cTnI). The FET-IGZO active channel was modified with PCN-Au NP materials, then a thiol-functionalized aptamer of cTnI was adsorbed into the active surface to create the biosensor. Source-drain current measurements were conducted to evaluate the Bio-FET sensor (Figure 1). We successfully demonstrate that this integrated system yields an output corresponding to the concentration of cTnI and then discuss the performance of the IGZO FET modified with PCN-Au NPs as a sensor for cardiac biomarkers.

## 2. Materials and Methods

### 2.1. Reagents and Instrumentation

Cardiac troponin C (cTnC), myoglobin, and cardiac troponin-I (cTnI) proteins were provided by ABCAM (U.K.). [ThiC6] CGT-GCAGTACGCCAACCT TTCTCATGCG CTGCCCCTTA (Tro4) is a thiol-modified Anti-cTnI DNA aptamer obtained from Sigma [31]. Mercaptohexanol (MCH), cholesterol, glucose, and human serum were purchased from Sigma. A 100 µM stock solution of the cTnI DNA aptamer was prepared in nuclease-free molecular biology-grade water and kept at −20 °C. Mercaptohexanol (MCH) stock solution (200 µM) was prepared in 10 mM PBS. Fisher BioReagents supplied the phosphate-buffered saline (PBS) tablets, which contain 2.7 mM KCl and 137 mM NaCl. Highly doped n-type Si (n++) wafers were purchased from Si-Mat (Germany). Oriented n-type-doped silicon wafers, which we utilized as the bottom-gate electrode, were obtained from Silicon Materials (Si-MAT). Testbourne Ltd., U.K., supplied the IGZO target (In_2_O_3_-Ga_2_O_3_-ZnO 1:1:2 mol %). Ultrapure water from a Milli-Q ultrapure system was used to prepare the aqueous solutions. Zeiss Merlin and TeneoVS field emission scanning electron microscopes and an FEI Themis Z were used to capture SEM and TEM images. The HORIBA EMAX X-ACT was used to record energy-dispersive X-ray spectroscopy (EDS) data. X-ray photoelectron spectroscopy (XPS) was carried out using anAMICUS XPS apparatus (Kratos Analytical, U.K.).

### 2.2. Synthesis of PCN-Au NPs

GCN was prepared from melamine via a typical thermal polymerization procedure. One gram of melamine was placed in a silica crucible and annealed at 550 °C for 4 h (heating rate: 5 °C/min). The resulting powder was washed three times with deionized water (DI) and ethanol to obtain GCN. Next, 10 mg/mL GCN dispersion was prepared in 100 mL H_2_SO_4_ and ultrasonically agitated until the yellow solution became colorless. The solution was then dispensed into 250 mL of ice-cold water to yield white-colored PCN. The product was separated and washed with water and ethanol. To prepare PCN-Au NPs, 0.5 g/mL aqueous PCN dispersion was first prepared in 50 mL of water to which 0.10 M HAuCl_4_ (1 mL) and 0.10 M NaBH_4_ (10 mL) were added and stirred for 1 h. Finally, the product was separated, washed, and dried to obtain PCN-Au NPs.

### 2.3. Fabrication of Transistor-Based Sensor Devices

IGZO FETs were fabricated via a bottom-up approach. The n++ Si wafers were procured from Silicon Materials (Si-Mat), and the device fabrication was carried out in the KAUST nanofabrication core labs. The silicon oxide that serves as the gate dielectric was grown using thermal oxidation, and IGZO was RF sputtered using a ceramic IGZO target. Organic and metal impurities were entirely eliminated from the Si wafers by dipping them in Piranha solution for 5 min. A native oxide etch was performed with buffered oxide etch solution. Then, silicon oxide (SiO_2_) with 150 nm thickness was deposited as a gate through a thermal oxidation procedure. Heavily doped n-type (n++) silicon wafers with thermally produced chlorinated SiO_2_ layers were employed to fabricate the bottom-gate bottom-contact FET devices. The wafer samples were cleaned using an ultrasonic cleaner for 5 min each in acetone and isopropyl alcohol (IPA), dehumidified for 5 min at 120 °C after being dipped in water, and briefly exposed to N_2_ gas. RF sputtering was utilized to deposit a thin film of IGZO with a thickness of 10 nm following our previously reported procedure [32]. Sputtering was carried out in the presence of Ar/O_2_ (20 SCCM/3 SCCM) plasma at a deposition pressure of 5 m Torr and 60 W RF power. Rapid thermal processing (RTP) at 5000 °C for 4 min in an O_2_ atmosphere was used to increase the stability of the FET device.

Lift-off deposition was utilized to deposit interdigitated top electrodes made of titanium (Ti) and gold (Au), then the interdigitated electrodes (IDEs) were fabricated by photolithography. DC magnetron sputtering at 400 W in the presence of Ar plasma was used to deposit metals, yielding Ti and Au with thicknesses of 10 nm and 100 nm, respectively. The IDE designs were fabricated using a typical photolithography procedure. The Si/SiO_2_ substrate was first spin-coated with AZ5214 photoresist, heated for 2 min at 110 °C, followed by 7 s exposure to UV light at 80 mJ/cm^2^ delivered through a mask plate. The substrate was immediately developed in AZ 726 developer for 1 min, then cleaned in DI water. The substrate was sealed in a black box and cured with N_2_ gas before exiting the yellow room. Radiofrequency (RF) sputter equipment was used to deposit Ti (10 nm)/Au (100 nm) on top of the as-prepared substrate. After a reasonable duration of sonication in acetone, the source and drain IDEs were obtained.

### 2.4. Fabrication of PCN-Au NP-Modified FET-IGZO

The dispersion of PCN-Au NPs (1.0 mg/mL) was prepared in 50% water/ethanol (*v*/*v*), and 1.0 µL of PCN-Au NPs was drop-casted onto the surface of the FET-IGZO devices and dried at 6 °C for 15 min.

### 2.5. Aptasensor Fabrication and Analysis

First, a mixture of 1 mM (optimized) cTnI aptamer and 20 mM MCH was prepared in 10 mM PBS (pH 7.4), then 1.0 µL of the aptamer-MCH mixture was dropped onto the active channel of the PCN-Au NP-modified FET-IGZO, incubated for 12 h, and the modified device was cleaned three times with water (pH 7.40). The device was subsequently treated with 0.10 mg/mL BSA for 30 min to prevent non-specific binding. The final device is a FET-IGZO PCN-Au NPs aptasensor. Various concentrations of cTnI (0.001, 1, 10, 10^2^, 10^3^ ng/mL) were prepared in 10 mM PBS (pH 7.40). The active channel of the aptasensors was coated with approximately 1.0 µL cTnI solution, incubated for 10 min to allow binding, and then the devices were cleaned with water to remove the unbound analyte.

### 2.6. Sensing and Electrical Measurements

A commercial Keithely 4200 semiconductor characterization system (SCS) was used to measure the electrical behavior of the FET-IGZO PCN-Au devices in the presence of various concentrations of cTnI. The Bio-FET device was biased by scanning the gate voltage (V_GS_) = −1.0 V to 20 V for various drain voltages (V_DS_) stepped from 5.0 V to 30 V, and the resultant transfer characteristics were measured. To measure the output characteristics, the drain voltage was scanned between 0 V and 8.0 V, and the resultant gate voltage was stepped at 8.0 V. The transfer and output properties of the device were measured at various concentrations of cTnI. All the analyses were performed at least three times, and the average of those results was utilized to make the figures.

## 3. Results and Discussion

### 3.1. Morphological and Structural Characterization of PCN-Au NP Materials

**Figure 2:**
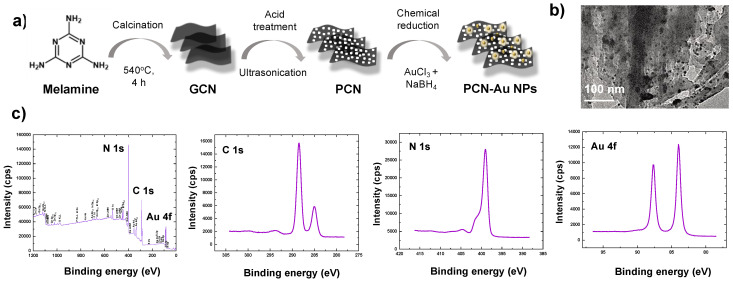
(**a**) Synthesis procedure for PCN-Au NPs, (**b**) TEM and (**c**) XPS analysis of PCN-Au NPs.

The synthesis route for PCN-Au NPs is shown in Figure 2a. Adding Au nanoparticles to the PCN surface further enhanced the conductivity and aptamer-immobilizing properties. Au NPs have distinct biosensing characteristics due to the formation of Au-S links, which display a strong binding affinity with thiolated aptamers [33]. The produced PCN-Au NPs were characterized using transmission electron microscopy (TEM) and X-ray photoelectron spectroscopy (XPS). TEM of the PCN-Au NPs revealed sheet structures of PCN with a homogeneous distribution of highly diffused Au nanoparticles (Figure 2b). The nanosheets are attached via bright solitary dots, which have an average size of roughly 5.0 nm. As shown in Figure 2c, sp^2^ hybridized -C-N = C, tertiary N (N-C), -C-N-H, and C-N bonds with binding energies of 398.2, 398.9, 399.8, and 400.8 eV, respectively, were observed, validating the structure of PCN [30]. Additionally, the high-resolution Au 4f spectra displayed peaks at 83.1 eV (4f_7/2_) and 87 eV (4f_5/2_), which demonstrates that Au was successfully loaded onto the PCN sheets, causing a localized fluctuation in PCN electron density and donation of electrons from PCN to the Au NPs [30]. N-defective sites were determined by computing the C/N elemental ratio from the survey scan XPS spectra. The C/N elemental ratio for PCN was 0.79. A higher C/N ratio of 0.89 was observed for PCN-Au NPs, which demonstrates the production of a high number of N-defective sites when Au is present.

### 3.2. Surface Morphology of FET-IGZO PCN-Au NPs (Bio-FET Functionalization)

**Figure 3:**
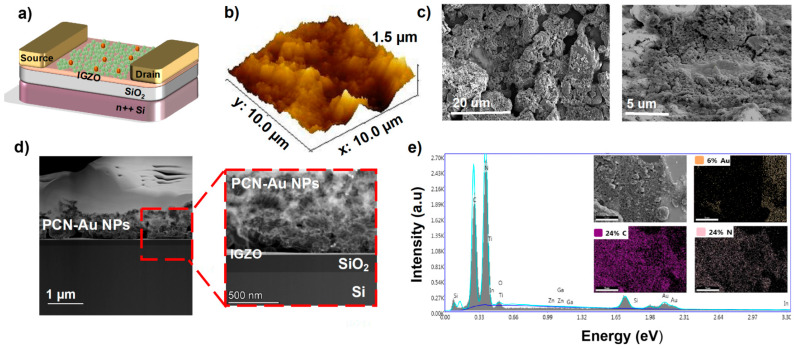
(**a**) Schematic of the fabricated PCN-Au NPs IGZO FET (cross-section) with PCN-Au NPs IGZO as the channel and the active sensing layer and Ti/Au as the source and drain electrodes of the transistor. (**b**) AFM image of PCN-Au NPs, which have a mean roughness of 1.47 nm. (**c**) Scanning electrode microscopy (SEM) images of the PCN-Au NPs IGZO FET channel. (**d**) TEM images of different layers of the device stack. (**e**) EDS for PCN-Au NPs IGZO FET.

A schematic illustration of the PCN-Au NP-based FET-IGZO sensor with the cTnI aptamer for cTnI protein detection is shown in Figure 3a. To enable aptamer conjugation, Au NPs were chemically functionalized on the PCN sheet drop-cast on top of the FET-IGZO sensor. Atomic force microscopy (AFM) was used to measure the thickness of the PCN-Au sheet in order to further characterize the FET device. Figure 3b maps the atomic profiles of the expected elements, i.e., In, Ga, Zn, O, and Si, that make up the top interface of the device. The IGZO-PCN-Au sheet has a thickness of roughly 1.47 nm, indicating a few-layer structure. SEM images of PCN-Au modified FET-IGZO are displayed in Figure 3c at two different magnifications. One piece of PCN sheet with an Au NP-passivated surface was observed to bridge the gap between the drain and source electrodes. The number of sites is increased, voids are embellished with nanostructures, and it appears that the surface area is increased. The TEM image clearly shows that the stack layers of the device were separate (Figure 3d). The Au NPs were visible after drop casting the PCN-Au NPs on top of FET-IGZO, which show bright “dots” of NPs. Both micro- and nano-sized (PCN-Au NPs) porous structures were observed on the new surface. Element mapping using EDX spectroscopy revealed that the IGZO interface could be distinguished from SiO_2_ and contained the elements in IGZO, carbon, nitrogen, and gold (Figure 3e).

### 3.3. Characterization of FET-IGZO

**Figure 4:**
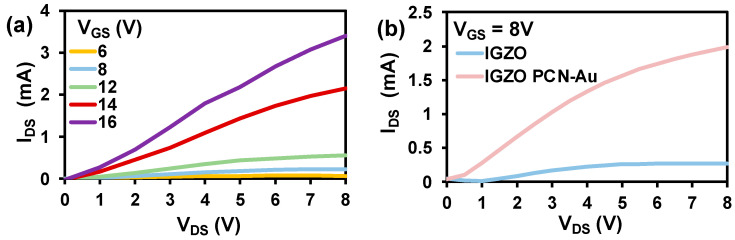
(**a**) Output characteristics of IGZO at varied bias voltages. (**b**) Output characteristics of IGZO and IGZO PCN-Au NPs at constant bias voltages.

A bottom-gate top-contact FET configuration was chosen to facilitate the functionalization of the top semiconducting IGZO channel with a biorecognition element. The output characteristics of the as-fabricated IGZO are displayed in Figure 4a. The device exhibits exceptional ON current in the order of mA and suitable gate control due to the high conductivity of the IGZO channel. Detailed analysis of the fabricated IGZO FET is reported in our previous paper [32]. PCN-Au NPs were chosen as the biorecognition element for fabrication of the Bio-FET, as adding Au nanoparticles to the PCN surface further enhances the conductivity and aptamer-immobilizing properties. Au nanoparticles have distinct biosensing characteristics due to the formation of Au-S links, which display a strong binding affinity with thiolated aptamers [33]. Hence, we drop-cast PCN-Au NPs onto fabricated IGZO FET. The additional layer of PCN-Au NPs on the IGZO FET led to an increase in the drain current of the Bio-FET (Figure 4b), indicating the enhanced conductivity of the device. The response of the sensor toward cTnI was tested using fixed V_gs_ (8 V) to investigate the performance of the sensor.

### 3.4. Sensing Performance

**Figure 5:**
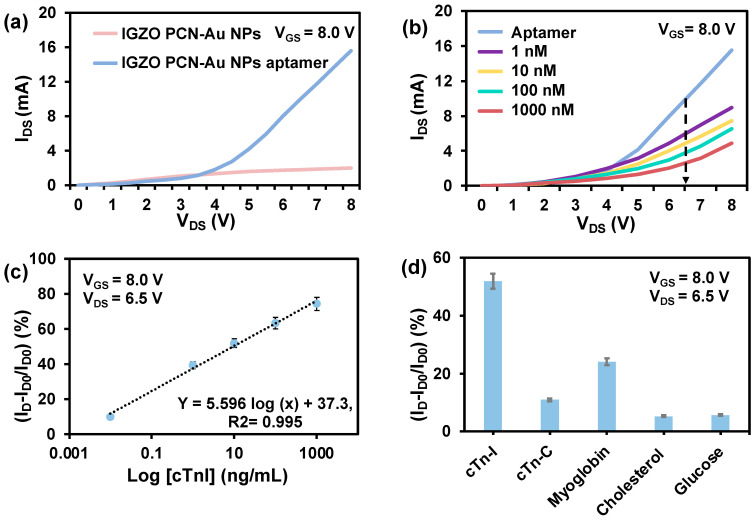
(**a**) Comparison of the aptamer output curves for IGZO PCN-Au NPs and IGZO PCN-Au NPs at constant bias voltage. (**b**) Detection of cTnI analyte at different concentrations (1.0 ng/mL to 1.0 μg/mL) using the Au/PCN-FET biosensor at sweep V_D_ of 0–8 V with V_SG_ = 15 V. (**c**) Calibration of the relative I_DS_ response curve of the biosensor at different cTnI concentrations, showing the LOD and sensitivity = Δ Drain Current/Δ cTnI conc. (g/mL) = slope of calibration curve, m. (**d**) Relative changes in I_DS_ for 10 ng/mL cTnC, myoglobin, cholesterol, and glucose (non-complementary control analytes) compared to 10 ng/mL of the target protein cTnI.

PCN-Au NPs Bio-FET was used to create an ultrasensitive cTnI sensor (Figure 5a). The PCN-Au Bio-FET sensing responses were analyzed at cTnI concentrations ranging from 0.01 ng/mL to 1000 ng/mL (Figure 5b). The positive and negative cTnI levels in the blood of patients who have suffered an AMI and blood samples from healthy patients were used to determine the concentration range. As the concentration of cTnI increased, the I_DS_ responses of the PCN-Au NPs Bio-FET sensor gradually decreased. This decrease results from the specific binding of the aptamer to the cTnI, which hinders electron transport. The hindrance varies depending on the amount of cTnI bound to the Bio-FET. The linear range of the calibration curve, which was obtained at V_DS_ = 6.50 V, was between 0.01 ng/mL and 1000 ng/mL (Figure 5c). The corresponding linear regression equation is ΔI = 5.596 log [cTnI] + 37.3; R^2^ = 0.995. The LOD was calculated to be 0.0066 ng/mL, and the sensitivity was 0.6 μA/(ng/mL), which covers the clinically significant range of cTnI in actual patient samples. The concentrations of cTnI range from 0 to 0.01 ng/mL in the blood of healthy patients, from 0.01 to 0.04 ng/mL in patients with mild myocarditis, and from 0.06 ng/mL or more in patients with necrotic myocardial injury [10]. The analytical performance of the PCN-Au NPs Bio-FET sensor outperforms or is on par with conventional bioassays in terms of linear range, detection limit, and sample volume (Table 1). The LOD is either better or comparable to the previous reports, and this highlights the potential of combining IGZO with PCN-Au NPs for the diagnosis of AMI. Another benefit is the use of a smaller sample volume, as the majority of other sensors require larger sample volumes. The majority of the technologies currently in use rely on conventional material coatings, but we used a simple drop casting method to produce FET-based sensors that are inexpensive, highly conductive, and have a high-quality 3D porous surface. Next, the selectivity of the Bio-FET was evaluated in the presence of several biomolecules that may interfere, such as cTnC, myoglobin, cholesterol, and glucose (Figure 5d). These molecules were selected on the basis of their structural similarity to cTnI. These other compounds exhibit noticeably weaker reactions than cTnI. The Bio-FET reaction to cTnI led to a 47% change in current, which is approximately times larger than the responses to cTnC, myoglobin, cholesterol, and glucose, respectively. The interference was less than 5%. These results indicate the high selectivity of FET-IGZO PCN-Au NPs, which is likely to be a result of the selectivity of cTnI-specific aptamers and successful immobilization of the cTnI aptamers on top of the PCN-Au NPs.

## 4. Conclusions

We successfully demonstrate a PCN-Au NPs and IGZO Bio-FET sensor for rapid, sensitive, quantitative monitoring of cardiac troponin biomarkers (cTnI). PCN-Au NPs offer a highly porous and enhanced surface area for the immobilization of aptamers. The PCN and IGZO materials are compatible with transistor device format and offer significant biosensing properties in terms of accommodating and providing microstructures for cTnI aptamers and enhancing the binding effectiveness of the cTnI with the transistor. The biosensing features of the Bio-FET (linear range: 0.01–1000 ng/mL) and detection limit 0.0066 ng/mL) are in a clinically useful range, suggesting that the biosensor could be used for practical applications. This sensor holds potential for the rapid, sensitive diagnosis of AMI. Future research is directed toward translating this device into a point-of-care device that can be used in non-laboratory settings.

## Figures and Tables

**Table 1:** Comparison of the analytical parameters of the IGZO PCN-Au NPs Bio-FET with previously reported FET biosensors.

S.No.	Sensing Materials	Biosensor Type	Receptor	LOD (ng/mL)	Detection Range (ng/mL)	Ref.
1.	^1^ ZnFe_2_O_4_/LSGE	Electrochemical	Aptamer	0.001	0.001–200	[11]
2.	^2^ MIP/BNQDs/GCE	Electrochemical	MIP	0.0005	0.01–5.0	[34]
3.	Graphene oxide	Fluorescence	Aptamer	0.07	0.10–6.0	[35]
4.	^3^ ZnO NPs/SOI wafer	FET	Antibody	0.0032	1–10,000	[36]
5.	^4^ AlGaN/GaN HEMT	FET	Antibody and aptamer	0.0026	0.006–148	[37]
6.	^5^ Au arrays/PDMS	^6^ EDL-FET	Antibody	-	0–24	[38]
7.	IGZO/PCN-Au NPs	FET	Aptamer	0.0066	0.01–1000	This work

^1^ ZnFe_2_O_4_/LSGE: zinc ferrite/laser-scribed graphene electrode; ^2^ MIP/BNQDs/GCE: molecularly imprinted polymer/boron nitride quantum dots/glassy carbon electrode; ^3^ ZnO NPs/SOI wafer: zinc oxide nanoparticles/silicon-on-insulator wafer; ^4^ AlGaN/GaN HEMT: aluminum gallium nitride high electron mobility transistor; ^5^ Au arrays/PDMS: gold arrays/polydimethylsiloxane; ^6^ EDL-FET: electric double-layer field effect transistor.

## Data Availability

Not applicable.

## References

[1] Vogel B., Acevedo M., Appelman Y., Merz C.N.B., Chieffo A., Figtree G.A., Guerrero M., Kunadian V., Lam C.S., Maas A.H. (2021). The Lancet women and cardiovascular disease Commission: Reducing the global burden by 2030. Lancet.

[2] Mani V., Durmus C., Khushaim W., Ferreira D.C., Timur S., Arduini F., Salama K.N. (2022). Multiplexed sensing techniques for cardiovascular disease biomarkers—A review. Biosens. Bioelectron..

[3] Countdown N. (2018). NCD Countdown 2030: Worldwide trends in non-communicable disease mortality and progress towards Sustainable Development Goal target 3.4. Lancet.

[4] Khushaim W., Peramaiah K., Beduk T., Vijjapu M.T., de Oliveira Filho J.I., Huang K.-W., Mani V., Salama K.N. (2022). Porous graphitic carbon nitrides integrated biosensor for sensitive detection of cardiac troponin I. Biosens. Bioelectron. X.

[5] Kaptoge S., Pennells L., De Bacquer D., Cooney M.T., Kavousi M., Stevens G., Riley L.M., Savin S., Khan T., Altay S. (2019). World Health Organization cardiovascular disease risk charts: Revised models to estimate risk in 21 global regions. Lancet Glob. Health.

[6] Khushaim W., Mani V., Peramaiya K., Huang K.-W., Salama K.N. (2022). Ruthenium and Nickel Molybdate-Decorated 2D Porous Graphitic Carbon Nitrides for Highly Sensitive Cardiac Troponin Biosensor. Biosensors.

[7] Gong F.F., Vaitenas I., Malaisrie S.C., Maganti K. (2021). Mechanical complications of acute myocardial infarction: A review. JAMA Cardiol..

[8] Surya S.G., Majhi S.M., Agarwal D.K., Lahcen A.A., Yuvaraja S., Chappanda K.N., Salama K.N. (2020). A label-free aptasensor FET based on Au nanoparticle decorated Co_3_O_4_ nanorods and a SWCNT layer for detection of cardiac troponin T protein. J. Mater. Chem. B.

[9] Anderson J.L., Morrow D.A. (2017). Acute myocardial infarction. N. Engl. J. Med..

[10] Szunerits S., Mishyn V., Grabowska I., Boukherroub R. (2019). Electrochemical cardiovascular platforms: Current state of the art and beyond. Biosens. Bioelectron..

[11] Rauf S., Mani V., Lahcen A.A., Yuvaraja S., Beduk T., Salama K.N. (2021). Binary transition metal oxide modified laser-scribed graphene electrochemical aptasensor for the accurate and sensitive screening of acute myocardial infarction. Electrochim. Acta.

[12] Sivashankar S., Sapsanis C., Buttner U., Salama K.N. (2015). Flexible low-cost cardiovascular risk marker biosensor for point-of-care applications. Electron. Lett..

[13] Ouyang M., Tu D., Tong L., Sarwar M., Bhimaraj A., Li C., Cote G.L., Di Carlo D. (2021). A review of biosensor technologies for blood biomarkers toward monitoring cardiovascular diseases at the point-of-care. Biosens. Bioelectron..

[14] Martínez-Domingo C., Conti S., De La Escosura-Muñiz A., Terés L., Merkoçi A., Ramon E. (2020). Organic-based field effect transistors for protein detection fabricated by inkjet-printing. Org. Electron..

[15] Yuvaraja S., Nawaz A., Liu Q., Dubal D., Surya S.G., Salama K.N., Sonar P. (2020). Organic field-effect transistor-based flexible sensors. Chem. Soc. Rev..

[16] Surya S.G., Raval H.N., Ahmad R., Sonar P., Salama K.N., Rao V.R. (2019). Organic field effect transistors (OFETs) in environmental sensing and health monitoring: A review. TrAC-Trends Anal. Chem..

[17] Sadighbayan D., Hasanzadeh M., Ghafar-Zadeh E. (2020). Biosensing based on field-effect transistors (FET): Recent progress and challenges. TrAC Trends Anal. Chem..

[18] Prakash M.D., Krsihna B.V., Satyanarayana B., Vignesh N.A., Panigrahy A.K., Ahmadsaidulu S. (2022). A study of an ultrasensitive label free silicon nanowire FET biosensor for cardiac troponin I detection. Silicon.

[19] Guo K., Wustoni S., Koklu A., Díaz-Galicia E., Moser M., Hama A., Alqahtani A.A., Ahmad A.N., Alhamlan F.S., Shuaib M. (2021). Rapid single-molecule detection of COVID-19 and MERS antigens via nanobody-functionalized organic electrochemical transistors. Nat. Biomed. Eng..

[20] Chae M.-S., Yoo Y.K., Kim J., Kim T.G., Hwang K.S. (2018). Graphene-based enzyme-modified field-effect transistor biosensor for monitoring drug effects in Alzheimer’s disease treatment. Sens. Actuators B Chem..

[21] Vijjapu M.T., Surya S.G., He J.-H., Salama K.N. (2021). Highly Selective Self-Powered Organic–Inorganic Hybrid Heterojunction of a Halide Perovskite and InGaZnO NO_2_ Sensor. ACS Appl. Mater. Interfaces.

[22] Vijjapu M.T., Surya S., Zalte M., Yuvaraja S., Baghini M.S., Salama K.N. (2021). Towards a low cost fully integrated IGZO TFT NO_2_ detection and quantification: A solution-processed approach. Sens. Actuators B Chem..

[23] Yang T.-H., Chen T.-Y., Wu N.-T., Chen Y.-T., Huang J.-J. (2017). IGZO-TFT biosensors for Epstein–Barr virus protein detection. IEEE Trans. Electron Devices.

[24] Kaczmarski J., Jankowska-Śliwińska J., Borysiewicz M.A. (2019). IGZO MESFET with enzyme-modified Schottky gate electrode for glucose sensing. Jpn. J. Appl. Phys..

[25] Lahcen A.A., Rauf S., Beduk T., Durmus C., Aljedaibi A., Timur S., Alshareef H.N., Amine A., Wolfbeis O.S., Salama K.N. (2020). Electrochemical sensors and biosensors using laser-derived graphene: A comprehensive review. Biosens. Bioelectron..

[26] Rajaji U., Chinnapaiyan S., Chen T.W., Chen S.M., Mani G., Mani V., Ali M.A., Al-Hemaid F.M.A., El-Shikh M.S. (2021). Rational construction of novel strontium hexaferrite decorated graphitic carbon nitrides for highly sensitive detection of neurotoxic organophosphate pesticide in fruits. Electrochim. Acta.

[27] Li Y., Li X., Zhang H., Xiang Q. (2020). Porous graphitic carbon nitride for solar photocatalytic applications. Nanoscale Horiz..

[28] Rajaji U., Chinnapaiyan S., Chen S.-M., Govindasamy M., Oliveira Filho J.I.d., Khushaim W., Mani V. (2021). Design and Fabrication of Yttrium Ferrite Garnet-Embedded Graphitic Carbon Nitride: A Sensitive Electrocatalyst for Smartphone-Enabled Point-of-Care Pesticide (Mesotrione) Analysis in Food Samples. ACS Appl. Mater. Interfaces.

[29] Mao S., Yu K., Chang J., Steeber D.A., Ocola L.E., Chen J. (2013). Direct growth of vertically-oriented graphene for field-effect transistor biosensor. Sci. Rep..

[30] Peramaiah K., Ramalingam V., Fu H.C., Alsabban M.M., Ahmad R., Cavallo L., Tung V., Huang K.W., He J.H. (2021). Optically and Electrocatalytically Decoupled Si Photocathodes with a Porous Carbon Nitride Catalyst for Nitrogen Reduction with Over 61.8% Faradaic Efficiency. Adv. Mater..

[31] Jo H., Gu H., Jeon W., Youn H., Her J., Kim S.-K., Lee J., Shin J.H., Ban C. (2015). Electrochemical aptasensor of cardiac troponin I for the early diagnosis of acute myocardial infarction. Anal. Chem..

[32] Vijjapu M.T., Surya S.G., Yuvaraja S., Zhang X., Alshareef H.N., Salama K.N. (2020). Fully Integrated Indium Gallium Zinc Oxide NO_2_ Gas Detector. ACS Sens..

[33] Oberhaus F.V., Frense D., Beckmann D. (2020). Immobilization techniques for aptamers on gold electrodes for the electrochemical detection of proteins: A Review. Biosensors.

[34] Yola M.L., Atar N. (2019). Development of cardiac troponin-I biosensor based on boron nitride quantum dots including molecularly imprinted polymer. Biosens. Bioelectron..

[35] Liu D., Lu X., Yang Y., Zhai Y., Zhang J., Li L. (2018). A novel fluorescent aptasensor for the highly sensitive and selective detection of cardiac troponin I based on a graphene oxide platform. Anal. Bioanal. Chem..

[36] Fathil M.F.M., Md Arshad M.K., Ruslinda A.R., Gopinath S.C.B., Nuzaihan M.N.M., Adzhri R., Hashim U., Lam H.Y. (2017). Substrate-gate coupling in ZnO-FET biosensor for cardiac troponin I detection. Sens. Actuators B Chem..

[37] Sarangadharan I., Regmi A., Chen Y.-W., Hsu C.-P., Chen P.-C., Chang W.-H., Lee G.-Y., Chyi J.-I., Shiesh S.-C., Lee G.-B. (2018). High sensitivity cardiac troponin I detection in physiological environment using AlGaN/GaN High Electron Mobility Transistor (HEMT) Biosensors. Biosens. Bioelectron..

[38] Sarangadharan I., Wang S.-L., Sukesan R., Chen P.-c., Dai T.-Y., Pulikkathodi A.K., Hsu C.-P., Chiang H.-H.K., Liu L.Y.-M., Wang Y.-L. (2018). Single Drop Whole Blood Diagnostics: Portable Biomedical Sensor for Cardiac Troponin I Detection. Anal. Chem..

